# Genotype, phenotype and treatment outcomes of 17 Malaysian patients with infantile-onset Pompe disease and the identification of 3 novel *GAA* variants

**DOI:** 10.1186/s13023-023-02848-6

**Published:** 2023-08-04

**Authors:** Mei-Yan Chan, Julaina Abdul Jalil, Yusnita Yakob, Siti Aishah Abdul Wahab, Ernie Zuraida Ali, Mohd Khairul Nizam Mohd Khalid, Huey-Yin Leong, Hui-Bein Chew, Jeya Bawani Sivabalakrishnan, Lock-Hock Ngu

**Affiliations:** 1 Department of Genetics, Hospital Kuala Lumpur, Ministry of Health Malaysia, Jalan Pahang, 50586 Kuala Lumpur, Malaysia; 2 Unit of Biochemistry, Institute for Medical Research, Ministry of Health Malaysia, Kuala Lumpur, Malaysia; 3 Unit of Molecular Diagnostics, Specialised Diagnostics Centre, National Institutes of Health, Ministry of Health Malaysia, Kuala Lumpur, Malaysia; 4 Unit of Inborn Errors of Metabolism and Genetic, Nutrition, Metabolism and Cardiovascular Research Centre, Institute for Medical Research, National Institutes of Health, Ministry of Health Malaysia, Kuala Lumpur, Malaysia; 5 Department of Pediatric Cardiology, Hospital Tunku Azizah, Ministry of Health Malaysia, Kuala Lumpur, Malaysia

**Keywords:** Acid alpha-glucosidase, Enzyme replacement therapy, *GAA*, Infantile-onset Pompe disease, Lysosomal storage disease

## Abstract

**Background:**

Pompe disease is a rare glycogen storage disorder caused by deficiency of the lysosomal enzyme acid alpha-glucosidase (GAA), leading to glycogen deposition in multiple tissues. Infantile-onset Pompe disease (IOPD) patients present within the first year of life with profound hypotonia and hypertrophic cardiomyopathy. Treatment with enzyme replacement therapy (ERT) has significantly improved survival for this otherwise lethal disorder. This study aims to describe the clinical and molecular spectrum of Malaysian IOPD patients, and to analyze their long term treatment outcomes.

**Methods:**

Seventeen patients diagnosed with IOPD between 2000 and 2020 were included in this retrospective cohort study. Clinical and biochemical data were collated and analyzed using descriptive statistics. GAA enzyme levels were performed on dried blood spots. Molecular analysis of the *GAA* gene was performed by polymerase chain reaction and Sanger sequencing. Structural modelling was used to predict the effect of the novel mutations on enzyme structure.

**Results:**

Our cohort had a median age of presentation of 3 months and median age of diagnosis of 6 months. Presenting features were hypertrophic cardiomyopathy (100%), respiratory insufficiency (94%), hypotonia (88%), failure to thrive (82%), feeding difficulties (76%), and hepatomegaly (76%). Fourteen different mutations in the GAA gene were identified, with three novel mutations, c.1552-14_1552-1del, exons 2–3 deletion and exons 6–10 deletion. The most common mutation identified was c.1935C > A p.(D645E), with an allele frequency of 33%. Sixteen patients received ERT at the median age of 7 months. Overall survival was 29%. Mean age of death was 17.5 months. Our longest surviving patient has atypical IOPD and is currently 20 years old.

**Conclusions:**

This is the first study to analyze the genotype and phenotype of Malaysian IOPD patients, and has identified the c.1935C > A p.(D645E) as the most common mutation. The three novel mutations reported in this study expands the mutation spectrum for IOPD. Our low survival rate underscores the importance of early diagnosis and treatment in achieving better treatment outcomes.

## Background

Pompe disease (OMIM #232300), also known as glycogen storage disease type II, is an autosomal recessive lysosomal storage disorder. Mutations in the *GAA* gene cause deficiency of the lysosomal enzyme acid alpha-glucosidase (GAA), leading to glycogen deposition in multiple tissues [1]. Pompe disease can be classified into the infantile and late-onset forms based on the age of onset, severity of organ involvement and rate of progression [2].

Classic infantile-onset Pompe disease (IOPD) presents before the age of 12 months with rapidly progressive cardiomyopathy, hypotonia, feeding difficulties, and respiratory insufficiency. If untreated, this often leads to death before the age of 2 years from cardiorespiratory failure [3, 4]. Atypical (non-classic) IOPD patients also have disease onset below the age of 1 year, but have less rapid progression of disease manifestations and longer survival. The distinguishing feature between the classic and non-classic forms of IOPD is the less severe cardiomyopathy [5]. Late-onset Pompe disease (LOPD) presents at varying ages ranging from 1 year until adulthood. Presentation is usually with proximal muscle weakness, with a slower rate of progression compared to the infantile form. Cardiomyopathy is not a feature in LOPD.

Enzyme replacement therapy (ERT) with recombinant alglucosidase alpha (rhGAA) has significantly improved outcomes for patients with IOPD [6]. ERT has been shown to reverse cardiomyopathy, improve motor function and increase survival [7]. It is now known that very early treatment with ERT prior to the onset of irreversible muscle damage leads to better patient outcomes [8]. However response to ERT is variable, with suboptimal outcomes in some patients even when treatment is started early [9, 10]. Cross-reactive immunological material (CRIM) negative patients are unable to synthesize native GAA enzyme and develop high sustained titres of rhGAA antibodies [11]. They respond poorly to treatment, and require immunomodulation prior to the initiation of ERT [12]. For most patients CRIM status can be predicted based on their genotype [13].

The *GAA* gene is located at chromosome 17q25.2–25.3, spans approximately 20 kb and contains 20 exons. To date, 648 disease causing variants have been identified and are listed in the Pompe disease variant database (http://www.pompevariantdatabase.nl/ updated in 2020) [14]. All types of mutations have been described, with missense mutations being the most frequently reported. Some mutations are found in increased frequencies in particular geographical regions, for example the c.1935C > A p.(D645E) mutation in the South of China, c.2662G > T p.(E888*) mutation in the North of China, and the intronic c.-32-13T > G mutation in individuals of European descent [15].

This is the first study to document the clinical characteristics and molecular spectrum of Malaysian patients with IOPD. In this study we also analyzed the long term treatment outcomes of IOPD patients who were started on enzyme replacement therapy.

## Methods

Ethical approval was obtained from the Malaysian Medical Research and Ethics Committee prior to study initiation. Study data was collated from a retrospective review of the medical records of all patients diagnosed with IOPD between 2000 and 2020 who were referred to Hospital Kuala Lumpur, the national treatment center for Pompe disease in Malaysia.

Failure to thrive was defined as weight below the third centile for age and gender using standard WHO growth charts. Left ventricular mass index (LVMI) was calculated using the formula: 0.8 × {1.04[(LVIDd + PWTd + SWTd)^3^ − (LVIDd)^3^]} + 0.6/BSA [16]. LVMI normalization was defined as LVMI below the 95th centile for age and gender, according to the published age specific centiles [17]. The use of diuretics or angiotensin-converting enzyme (ACE) inhibitors prescribed by the paediatrician was indicative of clinically significant heart failure.

GAA enzyme levels were performed on dried blood spots (DBS). The method used at the Biochemistry Laboratory of the Institute for Medical Research was as described by Chamoles et al. [18] with slight modification. 3 mm DBS was eluted with 270µL of HPLC water. 30µL aliquots were transferred to 96-well black assay plate after 1 h of incubation. The test was performed by using the artificial substrate 4-methylumbelliferyl-α-d-glucopyranosidase (70 mM; Sigma-Aldrich) in 40 mM sodium acetate (NaCH_3_COO) buffer at pH 3.7 (Sigma-Aldrich) with or without the addition of 10 µL 0f 8 µM acarbose solution (Toronto Research Chemicals, North York, Canada). In addition, the assay was also performed at pH 7.0 (40 mM NaCH_3_COO buffer adjusted with hydrochloric acid or sodium hydroxide) to assess the quality of DBS. All tests were run in duplicate. After 20 h of incubation at 37 °C, 30 µL of DBS eluate that had been stored in 4 °C overnight was added to specific wells that served as blanks. We used a standard curve for 4-methylumbelliferone (25 mM; Sigma-Aldrich) and each plate contained a concentration of 16.1 µM which was used for the calculation of enzyme activity. Lastly, the reaction was stopped by the addition of 200 µL EDTA buffer (150 mM, pH 11.3; Sigma-Aldrich). The fluorescence was read on the Tecan Spectrafluor fluorometer (Durham, NC) with 355 nm excitation and 460 nm emission wavelengths. In addition to enzyme activity, the inhibition with acarbose in percent and ratio of activities between pH 3.8 with inhibition to pH 7 were calculated to aid in the diagnostic evaluation of each specimen. Acarbose was used as inhibitor of maltase glucoamylase (MGA), an isoenzyme of acid alpha glucosidase (GAA), producing net GAA. The enzyme activities were expressed as percentage of enzyme in the patients to the normal control. Patients 1, 2, 4 and 7 had their DBS GAA enzyme levels performed at SA Pathology Lab, Adelaide prior to the development of the technique in Malaysia, with units expressed in umol/hour/L.

Molecular analysis of the *GAA* gene was performed using genomic DNA extracted using standard procedures. The purity and concentration of extracted DNA was measured using NanoDrop Spectrophotometer. DNA amplification by polymerase chain reaction (PCR) was carried out to confirm the mutations in *GAA* gene (NM_000152.4), using specific forward and reverse primers flanking the 20 exons including the splice sites. Subsequently, bi-directional DNA sequencing was performed on a fluorescent Genetic Analyzer ABI 3500 (Applied Biosystems) using BigDye Terminator Cycle Sequencing V3.1 chemistry (Applied Biosystems, Foster City, CA, USA). DNA sequencing data was analyzed using SeqScape software version 3.0 (Applied Biosystems). Identified mutations were referenced with the Human Gene Mutation Database (HGMD) to evaluate its significance and to determine whether the mutation had been previously reported. Further bioinformatics analysis was conducted using prediction tools to predict the alteration either as polymorphism or disease causing. Multiplex ligation-dependent probe amplification (MLPA) analysis was carried out using the SALSA MLPA Probemix P453-A2 kit to investigate the presence of large deletions in *GAA* gene when no variant or only one heterozygous variant was identified by DNA sequencing. This method was utilized in the detection of exons 6–10 deletion. Deletion of exons 2–3 in Patient 3 was identified by using single nucleotide polymorphism (SNP) markers on SNP heterozygosity analysis performed in another laboratory.

Structural modelling was then performed to illustrate the effect of the novel mutations (exons 2–3 deletion and exons 6–10-deletion) on protein structure. The wild type (WT) crystal structure of the human GAA protein was retrieved from the protein data bank (RSCB-PDB) (PDB ID: 5NN4). To create the mutant models of exons 2–3 and exons 6–10 deletions, we first made the FASTA sequence files of the modified proteins, then performed model building by using the WT crystal structure as a template. The mutant models were built by using the automated modelling server Swiss-Model (https://swissmodel.expasy.org/). The quality of the WT and mutant models were validated using Errat [19], Procheck [20] and ProSA-web [21]. The molecular dynamics (MD) simulations were then performed using GROMACS package (version 2019.4) (http://www.gromacs.org) for model refinement [22]. The simulation was run with the AMBER99SB force field at 300 K and 0.1 M NaCl for 1000 ps. The models were then analyzed by comparing between WT and mutant structures. The visualization of structures were performed using PyMOL [23].

CRIM assay was performed for only one patient in our cohort. In the other 16 patients, CRIM status and the severity of the mutation was predicted based on data from the Erasmus Pompe database. (http://www.pompevariantdatabase.nl/ updated in 2020) [14].

## Results

Seventeen patients were diagnosed with IOPD between 2000 and 2020, consisting of 11 females (65%) and six males (35%). None of the patients had parental consanguinity. All our patients were diagnosed with classic IOPD except patients 1 and 2 who had atypical IOPD. Three patients were diagnosed through high risk screening following an affected sibling (patients 2, 12 and 15), whereas the others were diagnosed after developing symptoms. The sibling pairs in this cohort were patients 1 and 2, and patients 5 and 12.

### Clinical characteristics

Table 1 shows the clinical, enzymatic and molecular features of our patients at diagnosis.

**Table 1:** Clinical, enzymatic and molecular characteristics of Malaysian infantile-onset Pompe disease (IOPD) patients at diagnosis

	Ethnicity	*GAA* gene mutation		GAA enzyme activity (%)	Age at presentation (months)	Failure to thrive	Presenting features (^‡^)	CK levels (units/L)	Cardiomegaly	Ejection fraction	LVMI (g/m^2^)	Heart failure
		Allele 1	Allele 2									
1	Chinese	c.1082C > T p.(P361L)	c.2815_2816delGT p.(V939Lfs*78)	0^†^	12	Yes	1, 3	1792	No	50%	NA	No
2	Chinese	c.1082C > T p.(P361L)	c.2815_2816delGT p.(V939Lfs*78)	0^†^	10	Yes	1, 2, 3, 4	1035	No	42.4%	NA	No
3	Chinese	c.796C > T p.(P266S)	Exons 2–3 deletion	1.2%	3	Yes	1, 2, 3, 4, 5	NA	Yes	NA	NA	Yes
4	Indigenous	NA	NA	0.05^†^	5	Yes	1, 2, 3, 4, 5	515	Yes	NA	NA	Yes
5	Chinese	c.2024_2026delACA p.(N675del)	c.1935C > A p.(D645E)	NA	4	Yes	1, 2, 3, 4, 5	NA	Yes	NA	NA	Yes
6	Chinese	c.1843G > A p.(G615R)	c.1935C > A p.(D645E)	NA	6	Yes	1, 2, 3, 4, 5	NA	Yes	NA	NA	Yes
7	Chinese	c.1411_1414delGAGA p.(E471Pfs*5)	c.1935C > A p.(D645E)	< 0.1^†^	3	No	1, 2, 3, 4	NA	Yes	9.9%	485	Yes
8	Chinese	NA	NA	2.3%	3	Yes	1, 2, 3	672	Yes	66%	99.4	Yes
9	Chinese	c.1411_1414delGAGA p.(E471Pfs*5)	c.1935C > A p.(D645E)	NA	3	Yes	1, 2, 3, 4, 5	NA	Yes	NA	NA	Yes
10	Chinese	c.1843G > A p.(G615R)	c.2815_2816delGT p.(V939Lfs*78)	6.5%	2	Yes	1, 2, 3, 4, 5	NA	Yes	NA	NA	NA
11	Indian	c.1551 + 1G > A p.(?)	c.1561G > A p.(E521K)	9.4%	3	Yes	1, 2, 3, 4, 5	824	Yes	26.2%	342	Yes
12	Chinese	c.2024_2026delACA p.(N675del)	c.1935C > A p.(D645E)	5.3%	2	No	1, 2, 3, 4	909	Yes	81.6%	159	No
13	Chinese	c.1935C > A p.(D645E)	c.1935C > A p.(D645E)	2.0%	4	Yes	1, 2, 3, 4, 5	1100	Yes	28%	483	Yes
14	Indian	c.1A > G p.(0)	Exons 6–10 deletion	3.4%	6	Yes	1, 2, 3, 5	986	Yes	34.3%	233.4	Yes
15	Chinese	c.2662G > T p.(E888*)	c.1935C > A p.(D645E)	0.6%	4	No	1, 2, 3, 4, 5	847	Yes	33%	461	Yes
16	Chinese	c.1552-14_1552-11del	c.2662G > T p.(E888*)	2.1%	1	No	1, 2, 4	582	Yes	NA	210.4	Yes
17	Malay	c.1935C > A p.(D645E)	c.1935C > A p.(D645E)	1.7%	2	Yes	1, 2, 3, 4, 5	930	Yes	29%	NA	Yes

*CK* creatine kinase, *GAA* acid alpha-glucosidase, *IOPD* infantile-onset Pompe disease, *LVMI* left ventricular mass index, *NA* not available

^†^GAA activity in umol/h/L (Normal range 0.3–10.0 umol/h/L) GAA enzyme analysis performed at SA Pathology Lab, Adelaide, Australia

^‡^1-Hypertrophic cardiomyopathy, 2-Respiratory insufficiency, 3-Hypotonia, 4-Feeding difficulties, 5-Hepatomegaly

Clinical features documented at presentation were hypertrophic cardiomyopathy (100%), respiratory weakness (94%), hypotonia (88%), failure to thrive (82%), feeding difficulties (76%) and hepatomegaly (76%). Our cohort had a median age at presentation of 3 months. The median age of diagnosis was 6 months, and the median age of ERT initiation was 7 months.

All patients with classic IOPD had cardiomyopathy at presentation with median LVMI of 233.4 g/m^2^ (range 83.4–485 g/m^2^). 71% of our cohort received medications for heart failure. Cardiomegaly was seen in 14 patients, large QRS complexes in 11 patients and conduction abnormalities in four patients. Only six patients had short PR intervals.

### GAA enzyme activity

GAA activity level was available for 14 patients. The other three patients were diagnosed based on typical clinical presentation and the identification of biallelic pathogenic variants in the *GAA* gene. The median GAA enzyme activity of the 10 patients measured in percentage enzyme activity was 2.2% (range from 0.6 to 9.4%). (Table 1).

### Molecular characteristics

*GAA* gene mutation analysis was performed for 15 patients. Two unrelated patients were homozygous for the c.1935C > A p.(D645E) mutation. The other 13 were compound heterozygotes.

The mutations found in our patients comprise of 30 alleles with a total of 14 different mutations. The most common mutation identified was c.1935C > A p.(D645E), occurring in eight patients (10 alleles) with allele frequency of (33%). Out of the 30 alleles analysed, 16 were missense variants, three were nonsense variants, five were deletions causing frameshifts, two were small deletions, two were splicing variants, and two were multiple exon deletions.

Three novel mutations were identified, c.1552-14_1552-1del, deletion of exons 2–3 and deletion of exons 6–10. For the c.1552-14_1552-1del variant found in patient 16, compound heterozygous with c.2662G > T p.(E888*), parental testing confirmed that the two variants were in trans. Deletion of exons 2–3 in patient 3 was detected using SNP heterozygosity analysis of the *GAA* gene. Loss of heterozygosity was detected in the SNP markers in exons 2–3 which was inherited from his mother. His father was heterozygote for the c.796C > T p.(P266S) mutation. Deletion of exons 6–10 in patient 14 was confirmed by MLPA. Parental testing confirmed that the two variants (c.1A > G and exons 6–10 deletion) were on opposite chromosomes.

**Fig. 1:**
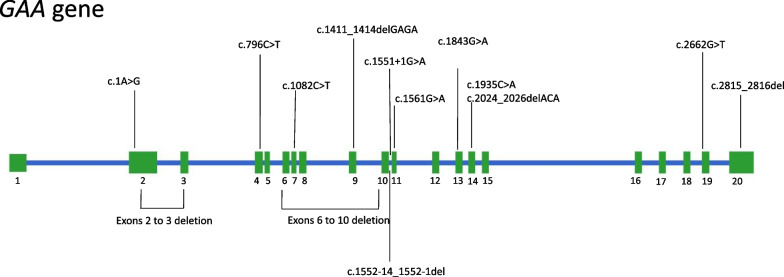
Mutation spectrum of 17 Malaysian Infantile-onset Pompe disease (IOPD) patients

The mutation spectrum of our patients are diagrammed in Fig. 1.

Previously described mutations are shown above, and novel mutations shown below the schematic *GAA* gene. The mutations are well distributed across the *GAA* gene.

### Structural analysis

**Fig. 2:**
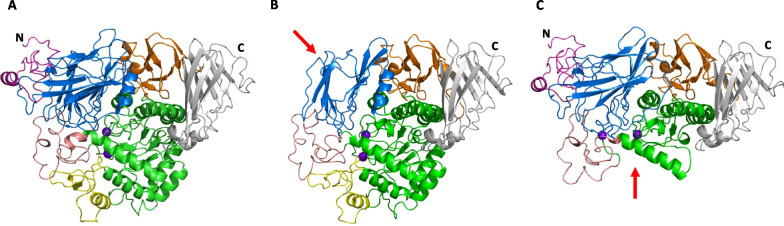
Models of wild type and mutant human GAA proteins, exons 2–3 and exons 6–10 deleted transcript variants with colored domains. **A** Structure of wild type GAA. **B** Structural model of GAA protein containing deletion of exons 2–3. Deletion of exons 2–3 results in the loss of the trefoil type-P domain and some missing β-sheets at the N-terminal β-sheet domain (indicated by a red arrow). **C** Structural model of GAA protein containing deletion of exons 6–10. Deletion of exons 6–10 results in some missing parts of the N-terminal β-sheet domain and the catalytic GH31 (β/α)_8_ barrel domain, including insert I and some parts of insert II (indicated by a red arrow). Cartoon representation of the structure of GAA consisting of the trefoil type-P domain (residues 81–136) (light magenta), the N-terminal β-sheet domain (residues 137–358) (marine), the catalytic GH31 (β/α)_8_ barrel domain (residues 359–720) (green) which consists of insert I (residues 444–492) (yellow) and insert II (residues 522–572) (salmon), the proximal β-sheet (residues 721–820) (orange) and distal β-sheet (residues 821–952) (grey) domains. Catalytic residues (purple) are depicted as spheres. *N* N terminus, *C* C terminus

The effects of the novel exonic deletion mutations on GAA structure were analyzed and shown in Fig. 2.

### Treatment with ERT and final outcome

Sixteen patients were started on enzyme replacement therapy. One patient declined treatment. Table 2 details their long term treatment outcomes. The longest treated patient has atypical IOPD and has received ERT for 15 years.

**Table 2:** Long term treatment outcomes for Malaysian infantile-onset Pompe disease (IOPD) patients

Pt	Predicted CRIM status*	Age ERT started (months)	Time lapse to ERT (months)	ERT regime (^†^)	Immunomodulation	Ig G antibody titer	LVMI normalization	Motor Achievement	Ventilation	Nasogastric or gastrostomy feeding	Final outcome	Current age/age of death
1	Positive	64	52	1	No	Negative	Yes	Walked unaided at 5 years	No	No	Alive	20 years
2	Positive	20	10	2	No	Negative	Yes	Walked unaided at 2 years 8 months	Nocturnal BiPAP	Gastrostomy	Alive	17 years
3	Positive	7	4	1	No	NA	NA	None	BiPAP	Nasogastric tube	Deceased	3.5 years
4	Unknown	11	6	2	No	NA	NA	None	Nasal oxygen	Nasogastric tube	Deceased	1 year
5	Positive	7	3	2	No	NA	NA	None	CPAP	Nasogastric tube	Deceased	8 months
6	Positive	9	3	2	No	NA	NA	None	CPAP	Nasogastric tube	Deceased	10 months
7	Positive	7	4	1	No	NA	No	None	BiPAP	Nasogastric tube	Deceased	2 years
8	Unknown	4	1	1	No	NA	No	Walked with support	No	No	Deceased	1 year 11 months
9	Positive	7	4	1	No	NA	NA	None	BiPAP	Nasogastric tube	Deceased	3 years
10	Positive	No ERT	NA	No ERT	NA	NA	No	None	Nasal oxygen	Nasogastric tube	Deceased	6 months
11	Positive	7	4	1	No	NA	No	Turned supine to prone	No	Nasogastric tube	Deceased	11 months
12	Positive CRIM assay	2	0	1	No	Negative	Yes	Walked unaided at 2 years 6 months	Nocturnal BiPAP	Gastrostomy	Alive	5 years 10 months
13	Positive	8	4	1	No	Negative	No	None	BiPAP	Nasogastric tube	Deceased	2 years
14	Unknown	7	1	2	Yes	1:3200	Yes	Walked unaided at 2 years 5 months	No	No	Alive	4 years 1 month
15	Positive	6	2	2	Yes	NA	No	None	CPAP, IPPV	Nasogastric tube	Deceased	7 months
16	Unknown	3	2	3	Yes	1:400	Yes	Walked unaided at 2 years 3 months	No	No	Alive	3 years 5 months
17	Positive	2.5	0.5	1	Yes	Negative	No	None	CPAP	Nasogastric tube	Deceased	6 months

*BiPAP* bilevel positive airway pressure, *CPAP* continuous positive airway pressure, *CRIM* cross-reactive immunological material, *ERT* enzyme replacement therapy, *IOPD* infantile-onset Pompe disease, *IPPV* invasive positive pressure ventilation, *LVMI* left ventricular mass index, *NA* not available

*CRIM status predicted based on Erasmus Pompe database (http://www.pompevariantdatabase.nl/ updated in Dec 2020) [14]

ERT regime ^†^1—Myozyme 20mg/kg 2 weekly, 2—Myozyme 20mg/kg weekly, 3—Myozyme 20mg/kg every 10 days

The median age of ERT initiation was 7 months. Twelve patients were started on ERT prior to the advent of immunomodulation. Four patients received immunomodulation according to the Duke University transient low-dose methotrexate protocol [24] in a treatment naive setting.

**Fig. 3:**
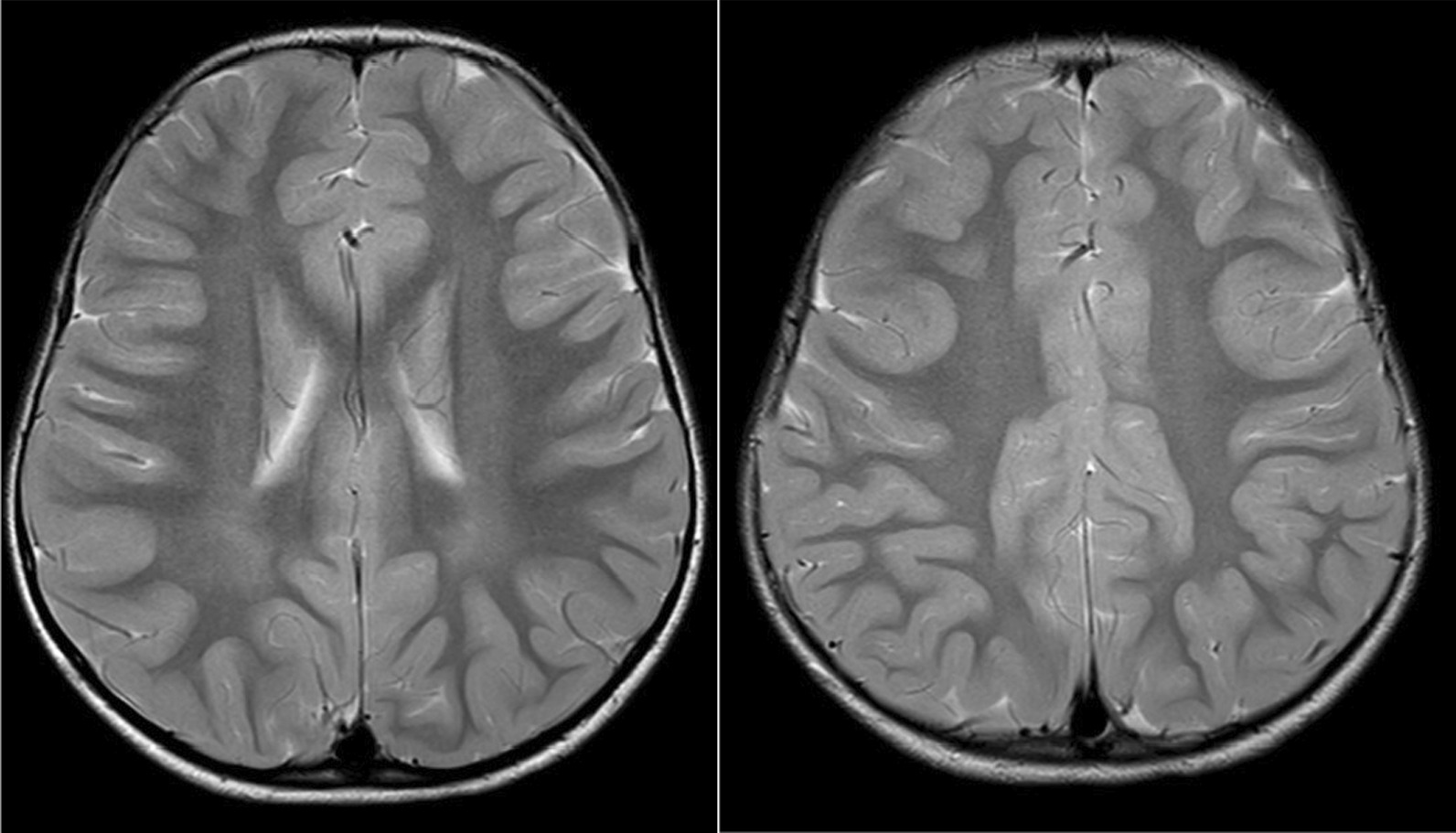
MRI brain of patient 14 taken at 3 years 8 months showing bilateral symmetrical T2 hyperintense changes in the periventricular white matter posteriorly

Five patients in our cohort are still alive at the time of writing, with current ages between 3 years 5 months and 20 years, giving an overall survival rate of 29%. Of the survivors, three have classic IOPD and two have atypical IOPD. Two siblings with atypical IOPD are now aged 17 years and 20 years respectively. All our surviving patients achieved independent ambulation between the ages of 2 years 3 months and 5 years. All achieved LVMI normalization following the initiation of ERT. The morbidities experienced by our surviving patients include kyphoscoliosis, restrictive lung disease, oropharyngeal dysfunction, speech delay and hearing impairment. MRI brain was performed for two patients in our cohort, patients 9 and 14. Both demonstrated symmetrical periventricular white matter T2 hyperintensities (Fig. 3).

The mean age of death for the 12 deceased patients was 17.5 months (range 6–42 months). Nine of the deceased patients were started on ERT at the age of 6 months and above.

## Discussion

The most frequently identified mutation in our cohort is c.1935C > A p.(D645E), with an allele frequency of 33%. This has also been reported to be the most common mutation in Southern China (25%), Taiwan (36%) and Thailand (32.4%) [25–27]. This mutation has been reported to cause both IOPD and LOPD [28, 29]. The Chinese patients in Malaysia share other common mutations with patients from China and Taiwan: c.2815_2816delGT p.(V939Lfs*78), c.2662G > T p.(E888*), c.1082C > T p.(P361L), c.1411_1414del p.(E471Pfs*5), and c.1843G > A p.(G615R) [25, 28, 29]. Malaysian Chinese have their ancestral roots in mainland China. This supports evidence of a founder effect for these common mutations.

To our knowledge the c.1552-14_1552-1del splice site mutation has not been reported in individuals with Pompe disease. This variant is not found in gnomAD exomes or genomes. This nucleotide substitution is predicted to disrupt the consensus splice site, cause aberrant splicing and subsequent loss of function.

Multiple exon deletions have been reported before in patients with Pompe disease. Deletion of exons 2–4 (c.148_859-11del) was reported in an Asian patient [30], and deletion of exons 8–15 (c.1195-18_2190-20del) in a Hispanic patient [31]. Both these patients were CRIM negative in a study by Bali et al. [13].

From structural analysis, the deletion of exons 2–3 results in the complete loss of signal peptide, propeptide and trefoil type-P domains as well as a partial loss of the N-terminal β-sheet domain [32]. In the absence of signal peptide, the co-translational transport of precursor enzyme into the lumen of the endoplasmic reticulum is impaired, leading to defective glycosylation and reduced enzyme activity [33].

Computational structural modelling revealed that the deletion of exons 6–10 may affect both the N-terminal β-sheet and the catalytic GH31 domains, which in the latter includes one of the key substrate-binding residues (D404) [34]. As noted in the wild type structure, the D404 residue is one of the five key residues (D404, D518, R600, D616 and H674) in the catalytic GH31 (β/α)_8_ barrel domain that plays a role in stabilizing the interaction between acid α-glucosidase and its substrate via hydrogen bonds [34]. Therefore, the loss of key substrate-binding residues due to the large multi-exonic deletion could weaken the enzymatic activity.

Our patients have a later median age of presentation and diagnosis compared to those in previous natural history studies. In the Dutch study by van de Hout et. al, the median age of presentation was 1.6 months and median age of diagnosis 5.3 months [3]. Kishnani et al. reported a median age of presentation of 2.0 months and median age of diagnosis 4.7 months [4]. The presenting features of respiratory difficulties, hypotonia, feeding difficulties and hepatomegaly are similar to those reported in previous cohorts [35]. Our survival rate of 29% is lower than the survival rates in previous cohorts, which range from 40 to 60% [9–11]. Contributing factors include late diagnosis and initiation of treatment.

Four of our patients share similar molecular mutations [two patients were homozygous for c.1935C > A p.(D645E), two patients were compound heterozygous for c.1411_1414del p.(E471Pfs*5) and c.1935C > A p.(D645E)] with patients in a study in Taiwan who were diagnosed via newborn screening and given very early ERT prior to symptom onset [8]. These patients achieved independent walking at normal ages (mean age 11.6 months) and had better developmental outcomes. In contrast, the four patients in our cohort passed away between the ages of 6 months and 3 years, and did not achieve any motor milestone. This highlights the importance of starting treatment early, prior to the onset of irreversible muscle damage. Newborn screening for Pompe disease is not available in Malaysia.

The long term morbidities experienced by the patients in our cohort are similar to those reported in previous studies, which include musculoskeletal, cardiac, respiratory, oropharyngeal, speech, hearing and neurocognitive dysfunction [36]. While there was a reversal of cardiomyopathy in all our surviving patients, residual skeletal muscle weakness was observed, with delayed attainment of motor milestones and subsequent gradual deterioration of function. This is consistent with the findings of other long term studies involving IOPD patients treated with ERT [37].

Several long term follow-up studies of IOPD patients on ERT have reported neuroimaging abnormalities, including ventricular enlargement, extra-axial cerebral fluid accumulation and delayed myelination, which resolved with time [38]. Periventricular white matter abnormalities have been reported in children with IOPD who were diagnosed through newborn screening and treated with very early ERT [39]. In addition, a characteristic pattern of white matter involvement which evolved with time has been observed, from periventricular to subcortical, and from superior to inferior [40]. At this point of time, the relationship of these neuroimaging findings to cognition remains unclear.

## Conclusions

This is the first study that analyzes the genotype and phenotype of IOPD patients in Malaysia, and has established the c.1935C > A p.(D645E) mutation as the most common mutation, with an allele frequency of 33%. The novel mutations identified in this study expands the mutation spectrum for IOPD. Our low survival rate of 29% highlights the importance of early diagnosis and initiation of treatment. Newborn screening for Pompe disease is the way forward to achieving better survival and long term outcomes.

## Data Availability

All data generated or analysed during this study are included in this published article [and its supplementary information files].

## Abbreviations

GAA: Acid alpha-glucosidase
IOPD: Infantile-onset Pompe disease
PCR: Polymerase chain reaction
ERT: Enzyme replacement therapy
LOPD: Late-onset Pompe disease
rhGAA: Recombinant human acid alpha-glucosidase
CRIM: Cross-reactive immunological material
WHO: World Health Organization
LVMI: Left ventricular mass index
BSA: Body surface area
ACE: Angiotensin-converting enzyme
DBS: Dried blood spot
MGA: Maltase glucoamylase
WT: Wild type
MD: Molecular dynamics
